# Supplementary material for: Improved Synthesis of a Novel Biodegradable Tunable Micellar Polymer Based on Partially Hydrogenated Poly(β-malic Acid-co-benzyl Malate)
Source: Molecules. 2021 Nov 26;26(23):7169. doi: 10.3390/molecules26237169 (PMC8658956; doi:10.3390/molecules26237169)
Supplement: Supplementary file 1 [file molecules-26-07169-s001.zip › molecules-1468575-supplementary.pdf]

Supporting Information

Improved Synthesis of a Novel Biodegradable Tunable Micellar Polymer Based on Partially Hydrogenated Poly(β-malic acid-co-benzyl malate)

Zhe Yu [1,†], Haozhe Ren [2,†], Yu Zhang [1,†], Youbei Qiao [1], Chaoli Wang [1], Tiehong Yang [1,*] and Hong Wu [1,*]

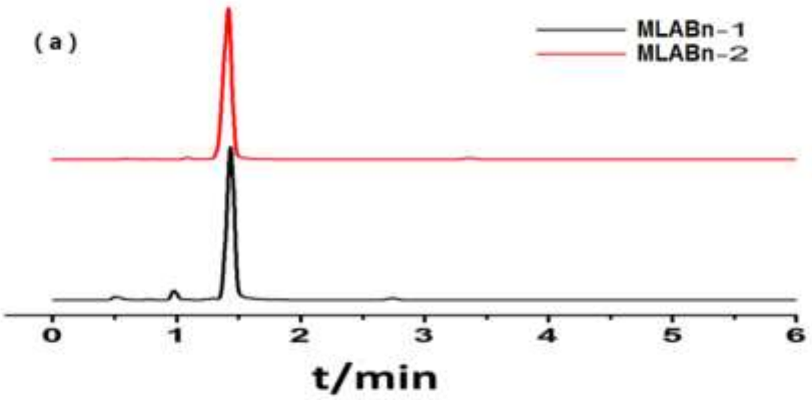

(b)

Sample	Retention time（min）	Peak area（%）
MLABn-1	1.436	90.2015
MLABn-2	1.418	96.2104

Figure S1 (a) HPLC of MLABn from aspartic acid or malic acid by C_{18} column. (b) Corresponding retention time and purity of MLABn.

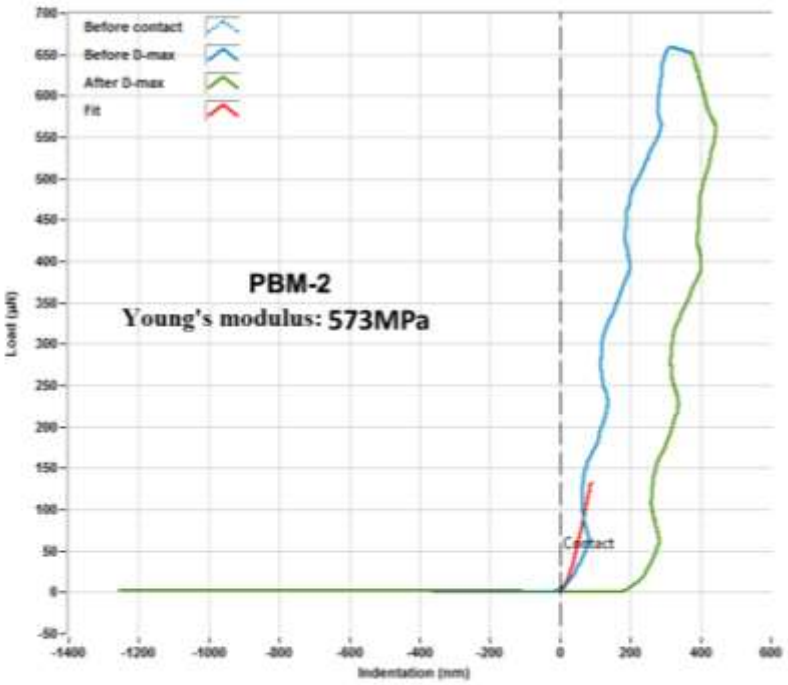

Figure S2 Young's modulus of PBM-2.

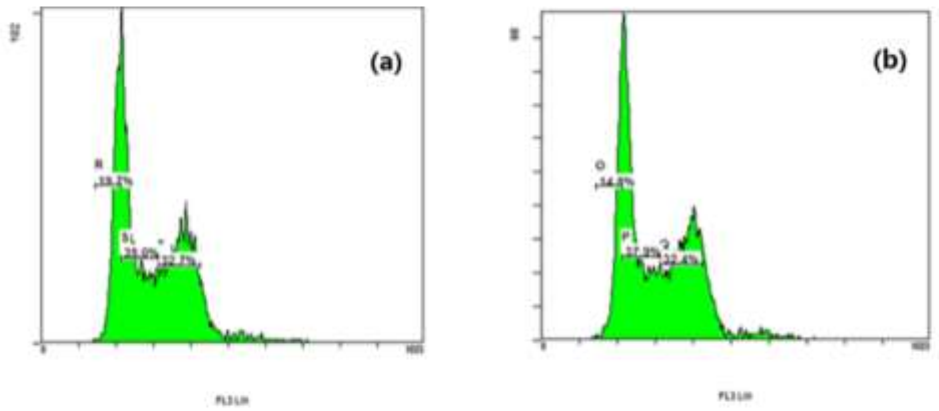

Region	Number	%Gated	X-Mean	X-CV
ALL	10563	100.00	311	37.00
R	2083	19.72	196	6.19
S	3692	34.95	247	12.03
T	3459	32.75	371	8.43

Region	Number	%Gated	X-Mean	X-CV
ALL	10525	100.00	319	36.46
O	1553	14.76	197	6.88
P	3986	37.87	246	11.76
Q	3409	32.39	374	8.56

Figure S3 Cell cycle analysis of PBM-2. (a) control group; (b) PBM-2.

Table S1. The effect of reaction condition on the yield of MLABn.

experiment	Reaction time (h)	Temperature (°C)	yield (%)
1	6	20	17%
2	12	20	39%
3	24	20	25%
4	12	0	--
5	12	35	14%
6	12	50	5%

Table S2. The optical rotation values of the reagents and products.

Sample	L-aspartic acid (c=0.5, 6N HCl)	L-malic acid (c=1, pyridine)	D-malic acid (c=1, pyridine)	MLABn-1 (c=1, dioxane)	MLABn-2 (c=1, dioxane)	MLABn-3 (c=1, dioxane)	PMLA-1 (c=0.5, water)	PMLA-2 (c=0.5, water)	PMLA-2 (c=0.5, water)
Optical rotation	+12.4	-27.6	+26.9	+0.024	+5.8	-8.5	-0.847	+69.9	-49.3

Table S3. The composition of C、H and O in PBM.

sample	C（%）	H（%）	O（%）
PBM (theoretical value)	64.0	4.9	31.1
PBM-1	62.7	4.9	32.4
PBM-2	63.7	5.0	31.3

Table S4. Molecular weight and related thermodynamic parameters of PBM.

Sample	Monomer/initiator (mol/mol)	polymerization time/d	Mη	Tg/°C	Tm/°C	polydispersity
PBM-1	1500:1	5	12000a	19	-	1.195
PBM-1	1500:1	-	6252b	-	-	
PBM-2	1500:1	5	10800	-	190	

a) The Mn of PMLABn-1 before degradation. b) The Mn of PBM-1 after degradation at 37 °C.
